# Usefulness of the artificial intelligence-mediated virtual chromoendoscopy in peroral cholangioscopy

**DOI:** 10.1055/a-2142-4555

**Published:** 2023-08-21

**Authors:** Ryosuke Sato, Kazuyuki Matsumoto, Hideaki Kinugasa, Daisuke Uchida, Shigeru Horiguchi, Hironari Kato, Motoyuki Otsuka

**Affiliations:** Department of Gastroenterology and Hepatology, Okayama University Hospital, Okayama, Japan

Chromoendoscopy is mainly used in gastrointestinal endoscopy to enhance the visibility of mucosal abnormalities in the digestive tract, by applying stains to the mucosa during endoscopy. The most commonly used stain in chromoendoscopy is indigo carmine, which can highlight subtle changes when sprayed onto the mucosa. Indigo carmine staining is also expected to be useful for the diagnosis of biliary diseases on peroral cholangioscopy (POCS). However, because the bile duct lumen is usually filled with bile or saline during POCS, it is difficult to spray the indigo carmine. In addition, the safety of spraying indigo carmine in the bile duct is unclear.

This report presents the first case demonstrating the usefulness of virtual indigo carmine chromoendoscopy (VIC) on POCS using a “cycle-consistent adversarial network” (CycleGAN), an artificial intelligence technology which can convert white-light images to VIC images. VIC makes it easier to recognize the margins and surface irregularities of malignant lesions on POCS. 

A 74-year-old man was admitted to our hospital with jaundice. Computed tomography showed wall thickening at the distal bile duct. Endoscopic retrograde cholangiopancreatography showed a main lesion at the distal bile duct extending into the left hepatic duct ( Fig. 1 ). We therefore performed POCS to determine the extent of the resection required. POCS showed stenosis of the entire circumference at the distal bile duct ( Fig. 2 ). An elevated lesion with an irregular surface structure at the hepatic bifurcation ( Fig. 3 , Fig. 4 ) and a flat elevated lesion at the root of the right hepatic duct ( Fig. 5 ) were observed. CycleGAN generated VIC images, which made it easier to recognize clearly the surface irregularity of the lesion at the hepatic bifurcation ( Fig. 3 , Fig. 4 ) and the margin of the flat elevated lesion ( Fig. 5 , Video 1 ). Both lesions proved to be malignant on biopsy. Left hepatectomy and pancreatoduodenectomy were performed, and R0 resection was achieved.

**Fig. 1:**
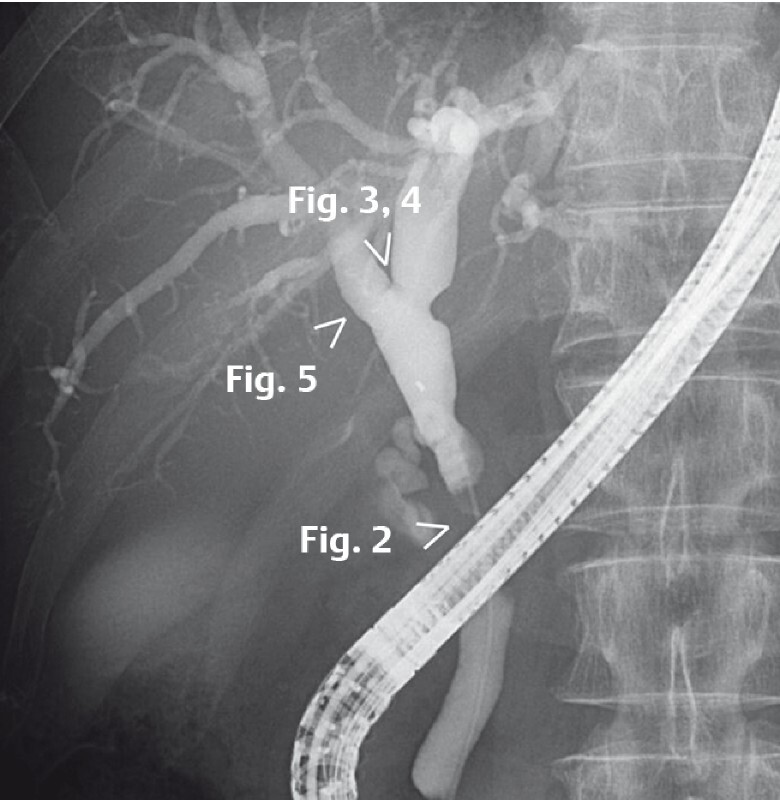
Endoscopic retrograde cholangiopancreatography showed a main lesion at the distal bile duct extending into the left hepatic duct. We performed peroral cholangioscopy (POCS) to determine the extent of the resection required.

**Fig. 2:**
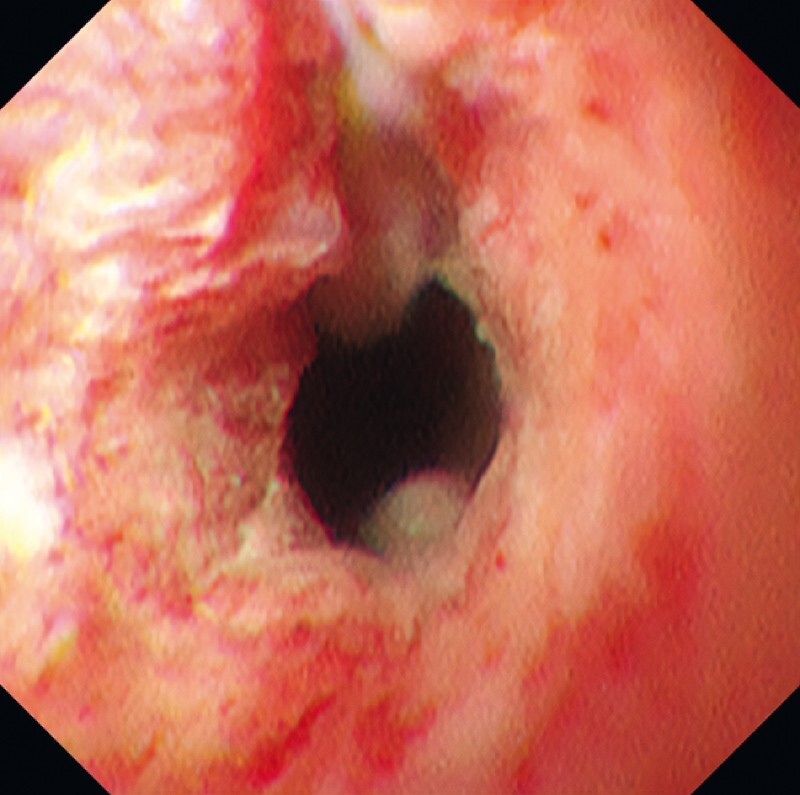
POCS showed stenosis of the entire circumference at the distal bile duct.

**Fig. 3:**
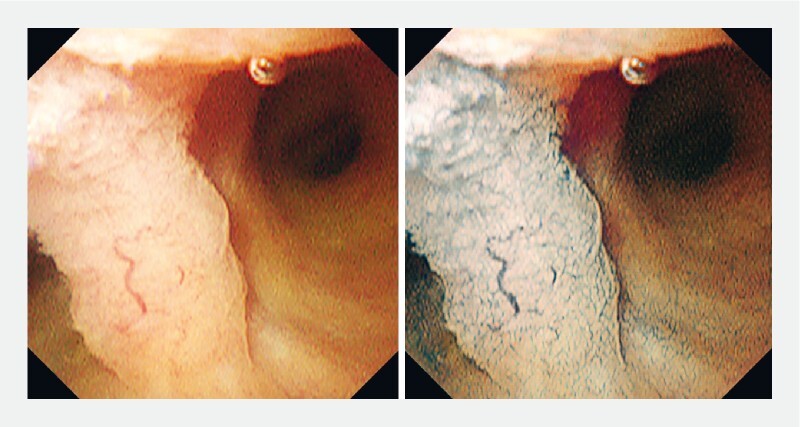
An elevated lesion with an irregular surface structure at the hepatic bifurcation was observed. Virtual indigo carmine chromoendoscopy (VIC) makes it easier to recognize the surface irregularities of malignant lesions.

**Fig. 4:**
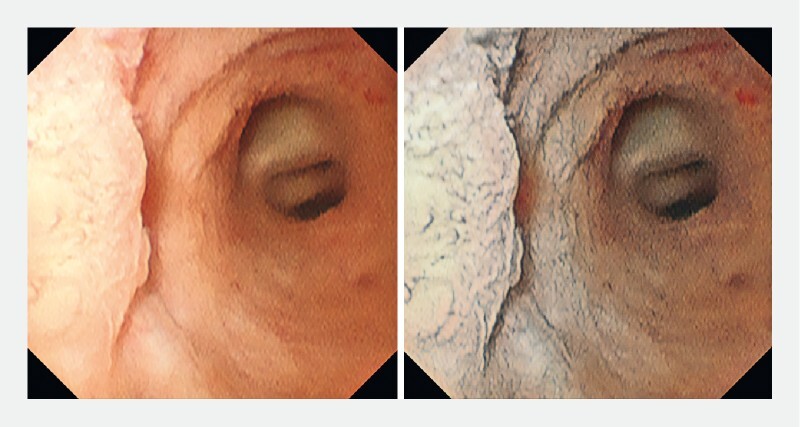
VIC made it easy to recognize the margin of the lesion, and the surface structure of the benign area also could be observed clearly.

**Fig. 5:**
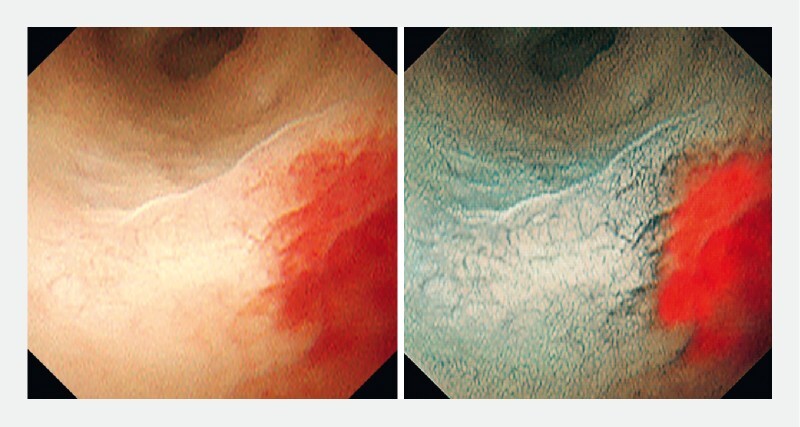
With VIC, it was also possible to observe clearly the margin of a flat elevated lesion at the root of the right hepatic duct.

**Video 1** Virtual indigo carmine chromoendoscopy (VIC) on peroral cholangioscopy using a “cycle-consistent adversarial network” (CycleGAN), an artificial intelligence technology which can convert white-light images to VIC images.


Endoscopy_UCTN_Code_CCL_1AZ_2AI

## Notes

Endoscopy E-Videos

https://eref.thieme.de/e-videos
*E-Videos* is an open access online section of the journal *Endoscopy* , reporting on interesting cases and new techniques in gastroenterological endoscopy. All papers include a high-quality video and are published with a Creative Commons CC-BY license. Endoscopy E-Videos qualify for HINARI discounts and waivers and eligibility is automatically checked during the submission process. We grant 100% waivers to articles whose corresponding authors are based in Group A countries and 50% waivers to those who are based in Group B countries as classified by Research4Life (see: https://www.research4life.org/access/eligibility/ ). This section has its own submission website at https://mc.manuscriptcentral.com/e-videos

